# Catatonia: Development of a neuropsychiatric entanglement through a clinical case

**DOI:** 10.1192/j.eurpsy.2023.2291

**Published:** 2023-07-19

**Authors:** M. Chtibi, H. Zarouf, H. Berrada, S. Belbachir, A. Ouanass

**Affiliations:** Arrazi University Psychiatric Hospital of Salé, Faculty of Medicine and Pharmacy of Rabat, Rabat, Morocco

## Abstract

**Introduction:**

Catatonia is a transnosographic and potentially fatal syndrome, most often associated with mood disorders or schizophrenia, but can also develop in autistic disorders, dementia, as well as in general medical conditions such as epilepsy, autoimmune encephalitis, hypercalcemia, hepatic encephalopathy, or diabetic ketoacidosis.

**Objectives:**

the objective is to understand the semiology and treatment of catatonic syndrome in a clinical case

**Methods:**

Clinical case

**Results:**

The work we present is based on a clinical case of a patient with schizophrenia presenting a catatonic syndrome, of which a neurological cause was first evoked but after clinical investigations the diagnosis of schizophrenia was retained and currently the patient is stabilized on Clozapine. It is imperative to recognize a catatonic syndrome in order to treat it quickly, as some of the etiologies that cause this syndrome and the consequences of the syndrome itself can be life-threatening.

**Conclusions:**

Catatonia remains a subject of research for centuries, the diagnosis is clinical, based on a set of criteria grouped in the DSM5, its etiologies are psychiatric and organic including neurological. Rapid diagnostic and therapeutic management is essential to avoid life-threatening complications.

**Disclosure of Interest:**

None Declared

